# Attenuation of Hyperlipidemia- and Diabetes-Induced Early-Stage Apoptosis and Late-Stage Renal Dysfunction via Administration of Fibroblast Growth Factor-21 Is Associated with Suppression of Renal Inflammation

**DOI:** 10.1371/journal.pone.0082275

**Published:** 2013-12-09

**Authors:** Chi Zhang, Minglong Shao, Hong Yang, Liangmiao Chen, Lechu Yu, Weitao Cong, Haishan Tian, Fangfang Zhang, Peng Cheng, Litai Jin, Yi Tan, Xiaokun Li, Lu Cai, Xuemian Lu

**Affiliations:** 1 Chinese-American Research Institute for Diabetic Complications, Wenzhou Medical University, Wenzhou, Zhejiang, China; 2 Ruian Center of the Chinese-American Research Institute for Diabetic Complications, Third Affiliated Hospital of the Wenzhou Medical University, Wenzhou, Zhejiang, China; 3 School of Pharmacy, Wenzhou Medical University, Wenzhou, Zhejiang, China; 4 Kosair Children’s Hospital Research Institute at the Department of Pediatrics, University of Louisville, Louisville, Kentucky, United States of America; University of Western Ontario, Canada

## Abstract

**Background:**

Lipotoxicity is a key feature of the pathogenesis of diabetic kidney disease, and is attributed to excessive lipid accumulation (hyperlipidemia). Increasing evidence suggests that fibroblast growth factor (FGF)21 has a crucial role in lipid metabolism under diabetic conditions.

**Objective:**

The present study investigated whether FGF21 can prevent hyperlipidemia- or diabetes-induced renal damage, and if so, the possible mechanism.

**Methods:**

Mice were injected with free fatty acids (FFAs, 10 mg/10 g body weight) or streptozotocin (150 mg/kg) to establish a lipotoxic model or type 1 diabetic model, respectively. Simultaneously the mice were treated with FGF21 (100 µg/kg) for 10 or 80 days. The kidney weight-to-tibia length ratio and renal function were assessed. Systematic and renal lipid levels were detected by ELISA and Oil Red O staining. Renal apoptosis was examined by TUNEL assay. Inflammation, oxidative stress, and fibrosis were assessed by Western blot.

**Results:**

Acute FFA administration and chronic diabetes were associated with lower kidney-to-tibia length ratio, higher lipid levels, severe renal apoptosis and renal dysfunction. Obvious inflammation, oxidative stress and fibrosis also observed in the kidney of both mice models. Deletion of the fgf21 gene further enhanced the above pathological changes, which were significantly prevented by administration of exogenous FGF21.

**Conclusion:**

These results suggest that FFA administration and diabetes induced renal damage, which was further enhanced in FGF21 knock-out mice. Administration of FGF21 significantly prevented both FFA- and diabetes-induced renal damage partially by decreasing renal lipid accumulation and suppressing inflammation, oxidative stress, and fibrosis.

## Introduction

Diabetes mellitus is a fatal disease whose incidence is increasing rapidly worldwide [1]. Complications associated with diabetes can be severe, and include diabetic kidney disease (DKD). Each diabetic patient has as much as a 40% lifetime risk of developing DKD, and it is the single most common cause of end-stage renal disease and diabetic nephropathy [2]. DKD begins as an early renal response to the acute pathogenic stresses of diabetes [3]–[6]. In these early stages, lipotoxicity (the accumulation of lipid intermediates) is considered a key instigator of diabetic renal damage and dysfunction [7]–[12].

Renal lipotoxicity is characterized by excessive intracellular free fatty acids (FFAs), which leads to the accumulation of potentially toxic metabolites such as diacylglycerol and ceramides [13]. Renal injury induced by lipotoxicity occurs through several mechanisms, including the generation of reactive oxygen species and release of proinflammatory and pro-fibrotic factors [14], [15]. All of these interact and finally contribute to renal apoptosis and chronic tubule damage with subsequent renal dysfunction and nephropathy [16]. To prevent early-stage renal cell death and halt the further development of DKD, an appropriate therapy must be found to simultaneously suppress lipid accumulation, inflammation, oxidative stress, and fibrotic factors during the early stages of diabetes.

Fibroblast growth factor (FGF)21, a member of the FGF family, is a lipid metabolic regulator which has beneficial effects against dyslipidemia and lipotoxicity [17], [18]. There is increasing evidence that FGF21 is involved in suppression of inflammation, oxidative stress, and the fibrotic effect. For example, serum FGF21 levels were elevated under inflammatory conditions [19]. Another study also confirmed that a deficiency of FGF21 enhanced inflammation induced by isoproterenol and lipopolysaccharide via inhibition of NF-κB (nuclear factor kappa-light-chain-enhancer of activated B cells) [20]. The same study found that FGF21 was associated with an anti-oxidative effect in cardiac cells, as evidenced by suppression of reactive oxygen species production [20]. Results of a recent study suggest that important biofunctions of FGF21 include anti-fibrotic effects. FGF21 significantly prevented the type 2 diabetes-induced gene expression of pro-fibrotic cytokines, including type IV collagen, plasminogen activator inhibitor-1 (PAI-1) and transforming growth factor (TGF)-β1 in the kidney [21].

To date, the study of FGF21 has focused on its effects in liver and adipose tissue [22]; kidney has relatively low levels of FGF21 [23]. Recently we demonstrated a significant and positive association between serum FGF21 and the progression of renal disease, from early- to end-stage chronic kidney disease [24]. Other research groups have also reported a close association between FGF21 levels and renal dysfunction and insulin resistance in end-stage renal disease patients [25], [26]. Altogether, the evidence suggests that lipotoxicity is crucially involved in the development of DKD, associated as it is with the suppression of the pathological mechanisms of hyperlipidemia, inflammation, oxidative stress and fibrotic effects. The present study investigated whether FGF21 has a renal protective function under lipotoxic and diabetic conditions, and the possible protective mechanism.

## Materials and Methods

### Ethics Statement

This study was carried out in strict accordance with the recommendations of the Guide for the Care and Use of Laboratory Animals of the National Institutes of Health. The Institutional Animal Care and Use Committee of the University of Louisville (IACUC #: 09018, 10102) approved the protocol. All surgery was performed under anesthesia induced by intraperitoneal injection of 1.2% 2,2,2-tribromoethanol (Avertin) at a dose of 0.2 mL/10 g body weight and all efforts were made to minimize suffering.

### Experimental animals

Male friend virus B-type (FVB) mice, 9 weeks old (18–22 of body weight), were obtained from Jackson Laboratory (Bar Harbor, Maine). FGF21 knock-out (FGF21-KO) mice, 9 weeks old (18–22 of body weight) with a C57BL/6J genetic background were given as a gift from Dr. Steve Kliewer, University of Texas Southwestern Medical Center. Age-matched C57BL/6J mice for controls were obtained from Jackson Laboratory. All mice were housed at 22°C with a 12:12-h light-dark cycle with free access to rodent chow and tap water. Animals were kept under these conditions for 2 weeks before the experiments.

**Mouse model of hyperlipidemia.** FVB mice were given a daily intraperitoneal injection of bovine serum albumin (BSA) with FFAs (BSA-FA; A4503, Sigma-Aldrich, St. Louis, MO) or essentially FFA-free BSA (BSA; A6003, Sigma) at 10 mg/g body weight, or sham-injected with the same volume of saline (n  =  6). Albumin solutions were prepared using sterile saline (150 mM NaCl) as a diluent [27]. The albumin concentration of the solutions was assayed and solutions were diluted to 33%. The pH of the BSA-FA solution was 6.5, and that of the BSA solution was 6.9 [28]. The BSA preparations tested negative for endotoxin [29]. The BSA-FA and BSA treated mice were intraperitoneally given FGF21 (100 µg/kg, synthetized in our laboratory by gene engineering [30]) or the same volume saline as well as the FFA treatment for 10 days.

**Type 1 diabetic mouse model.** Type 1 diabetes was induced in FVB, C57BL/6J, and FGF21-KO mice with a single intraperitoneal injection of streptozotocin (STZ) at 150 mg/kg, since acute β cell damage due to STZ rapidly increases tissue lipid accumulation [31]. In addition, the mice treated with same dose STZ, which unsuccessfully developed hyperglycemia were used to identify whether diabetes, but STZ, was the only reason to cause the subsequent pathological changes. After hyperglycemia was diagnosed, diabetic mice were treated with or without FGF21 (n  =  6) for 10 days. Then the mice were euthanized after collection of blood plasma and 24-h serum urea.

### Measurements for renal function

Blood urea nitrogen (BioAssay Systems, Hayward, CA) were assayed in accordance with the manufacturers’ instructions in the kits. Mouse urine was collected before the animals were euthanized. Urine albumin (Bethyl Laboratories, Montgomery, TX) and urinary creatinine (BioAssay Systems) were measured in accordance with the manufacturers’ instructions. Urinary total protein-to-creatinine (PCR) and urinary albumin-to-creatinine ratio (ACR) were calculated.

### Plasma and renal triglyceride (TG) assay

Plasma TG concentrations were measured using a TG assay kit (Cayman Chemicals, Ann Arbor, MI). For the renal TG assay, mouse kidneys were homogenized in 1× phosphate buffer saline (PBS). Tissue lipids were extracted with methanol: chloroform (1:2), dried in an evaporating centrifuge, and resuspended in 1% Triton X-100. A colorimetric assessment of renal TG levels was carried out using Triglyceride Assay Reagent (Thermo Fisher Scientific). Values were normalized to protein in homogenate before extraction, determined by the Bradford assay (Bio-Rad Laboratories, Hercules, CA).

### Oil Red O staining for lipid accumulation

Cryosections from optimal cutting temperature medium (OCT)-embedded tissue samples of the kidney (10-mm thick) were fixed in 10% buffered formalin for 5 min at room temperature, stained with Oil Red O for 1 h, washed with 10% isopropanol, and then counterstained with hematoxylin (DAKO, Carpinteria, CA) for 30 s. A Nikon microscope (Nikon, Melville, NY) was used to capture the oil red O-stained tissue sections at 40× magnification.

### Terminal deoxynucleotidyl transferase-mediated dUTP nick end labeling (TUNEL) assay

Kidney tissue was fixed in 10% formalin and embedded in paraffin. Fixed kidney tissues were cut into 3-mm-thick blocks. The tissue blocks were embedded in paraffin and cut into 5-µm slices. After deparaffinization (using xylene and ethanol dilutions) and rehydration the sections were stained for TUNEL with an ApopTag Peroxidase In Situ Apoptosis Detection Kit (Chemicon, CA, USA), as described in previous studies [32]. Briefly, each slide was deparaffinized and rehydrated, and treated with proteinase K (20 mg/L) for 15 min. The endogenous peroxidase was inhibited with 3% hydrogen peroxide for 5 min, and then incubated with the TUNEL reaction mixture containing terminal deoxynucleotidyl transferase (TdT) and digoxigenin-11-dUTP for 1 h. The TdT reaction was carried out in a humidified chamber at 37°C, and then 3,3-diaminobenzidine chromogen was applied. Hematoxylin was used as counterstaining. For the negative control, TdT was omitted from the reaction mixture. Apoptotic cell death was quantitatively analyzed by counting the TUNEL-positive cells selected randomly from 10 fields, at 40× magnification. Results were presented as the number of TUNEL-positive cells per 10^3^ cells.

### Western blot assays

Western blot assays were performed as described previously [33]. Briefly, renal tissues were homogenized in lysis buffer. Proteins were collected by centrifuging at 12 000 ×*g* at 4 °C. A sample of total protein was subjected to electrophoresis in a 10% SDS-PAGE gel. After electrophoresis of the gel and transferring the proteins to a nitrocellulose membrane, these membranes were rinsed briefly in tris-buffered saline, blocked in blocking buffer (5% milk and 0.5% BSA) for 1 h, and washed three times with tris-buffered saline containing 0.05% Tween 20 (TBST). The membranes were incubated serially with different primary antibodies (below) overnight at 4 °C, washed with TBST and incubated with secondary horseradish peroxidase–conjugated antibody for 1 h at room temperature. Antigen-antibody complexes were then visualized using an enhanced chemiluminescence kit (Amersham, Piscataway, NJ). The intensity of protein bands on blots was quantified by densitometric scanning (Epson perfection V700 photo, Epson) and analyzed by Quantity One software (Bio Rad).

The primary antibodies were against 3-nitrotyrosine (3-NT; 1∶1000, Chemicon), 4-hydroxynonenal (4-HNE; 1∶2000; Calbiochem, San Diego, CA), intercellular adhesion molecule-1 (ICAM-1; 1∶500; Santa Cruz Biotechnology, Santa Cruz, CA), anti-connective tissue growth factor (CTGF), β-actin (1∶1000; Santa Cruz), plasminogen activator inhibitor type 1 (PAI-1; 1∶2000, BD Biosciences, Sparks, MD), and tumor necrosis factor (TNF-α, 1∶500, Cell Signaling Technology, Danvers, MA).

### Statistical analyses

Data were collected from 6 mice per group and presented as mean ± standard deviation (SD). One-way ANOVA was used to determine general differences, followed by a post-hoc Tukey’s test for the difference between groups, using Origin 7.5 software for laboratory data analysis and graphing. Statistical significance was considered *P* < 0.05.

## Results

### FGF21 prevented hyperlipidemia-induced renal damage

**FGF21 prevented acute hyperlipidemia-induced renal dysfunction.** FVB mice were injected intraperitoneally BSA-FA (10 mg/g) with or without simultaneous administration of FGF21 (100 µg/kg) for 10 days (Table 1). The ratio of kidney weight to tibia length (KW/TL) and renal function were calculated. The results showed that the mean KW/TL was significantly higher in the mice of the BSA-FA group than in the BSA and control groups. In addition, FFA induced renal dysfunction characterized by higher ratio of urinary total protein to creatinine (PCR), and the ratio of urinary albumin-to-creatinine ratio (ACR). Although BUN of both BSA and BSA-FA treated mice tended to be higher compared to the mice in control group, the difference were not statistically significant respectively (Table 1). The rises of BUN, PCR and ACR were further enhanced inBSA-FFA treated mice. In contrast, administration of FGF21 significantly prevented lipotoxicity induced kidney weight increase (a feature of renal hypertrophy) and renal dysfunction (Table 1).

**Table 1:** Effect of FGF21 on lipotoxicity-induced renal hypertrophy and dysfunction.

	Control	FGF21	BSA	BSA-FA	BSA-FA/FGF21
KW/TL (mg/mm)	17.55±3.53	17.64±2.71	21.42±5.53	23.97±2.13 *	18.98±2.57 $
PCR (mg/mg)	24.45±3.52	25.56±4.37	79.33±6.23*	247.12±12.21 *#	114.76±9.26 $
BUN (mg/dl)	34.11 ±5.67	37.83±7.36	45.33±16.23	52.18±15.49	37.16±6.25
ACR (µg/mg)	107.43±11.23	116.58±12.35	16471.76±179.11*	36188.34±198.97 *#	10079.21±106.72$

* *P* < 0.05 compared with control; # *P* < 0.05 compared with BSA group; $ *P* < 0.05 compared with BSA-FA group.

**Figure 1:**
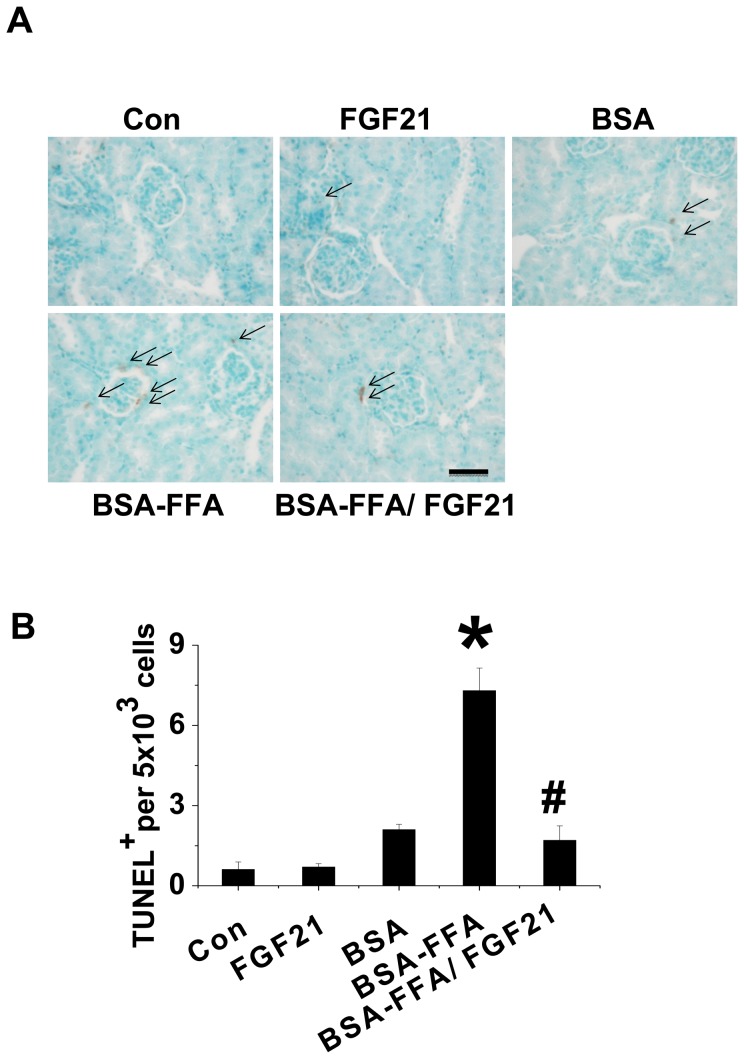
FGF21 prevents FFA-induced renal apoptosis. FVB mice were intraperitoneal injection with FFA (10 mg/g) with and without FGF21 (100 µg/kg) for 10 days. Renal apoptosis was examined with TUNEL staining (A) and semi-quantitative analysis for apoptotic examination was scored (B). Data are presented as mean ± SD (n  =  6 at least in each group). * *P* < 0.05 compared with control. ^#^ *P* < 0.05 compared with BSA-FFA.

**FGF21 prevented lipotoxicity induced renal apoptosis.** Renal dysfunction always initiates with apoptosis, which can be caused by lipotoxicity [16]. We tried to identify whether FGF21 had a protective effect against renal apoptosis upon injection of excess lipid. TUNEL staining showed larger numbers of apoptotic cells in the BSA-FA treated mice but not in either the FGF21 or BSA groups (Fig. 1A, B). However, administration of FGF21 significantly prevented FFA-induced renal apoptosis. In addition, the combined results of 3 independent experiments observing TUNEL-positive cells revealed that FGF21 almost completely prevented FFA-induced renal apoptosis (Figure 1B).

**Figure 2:**
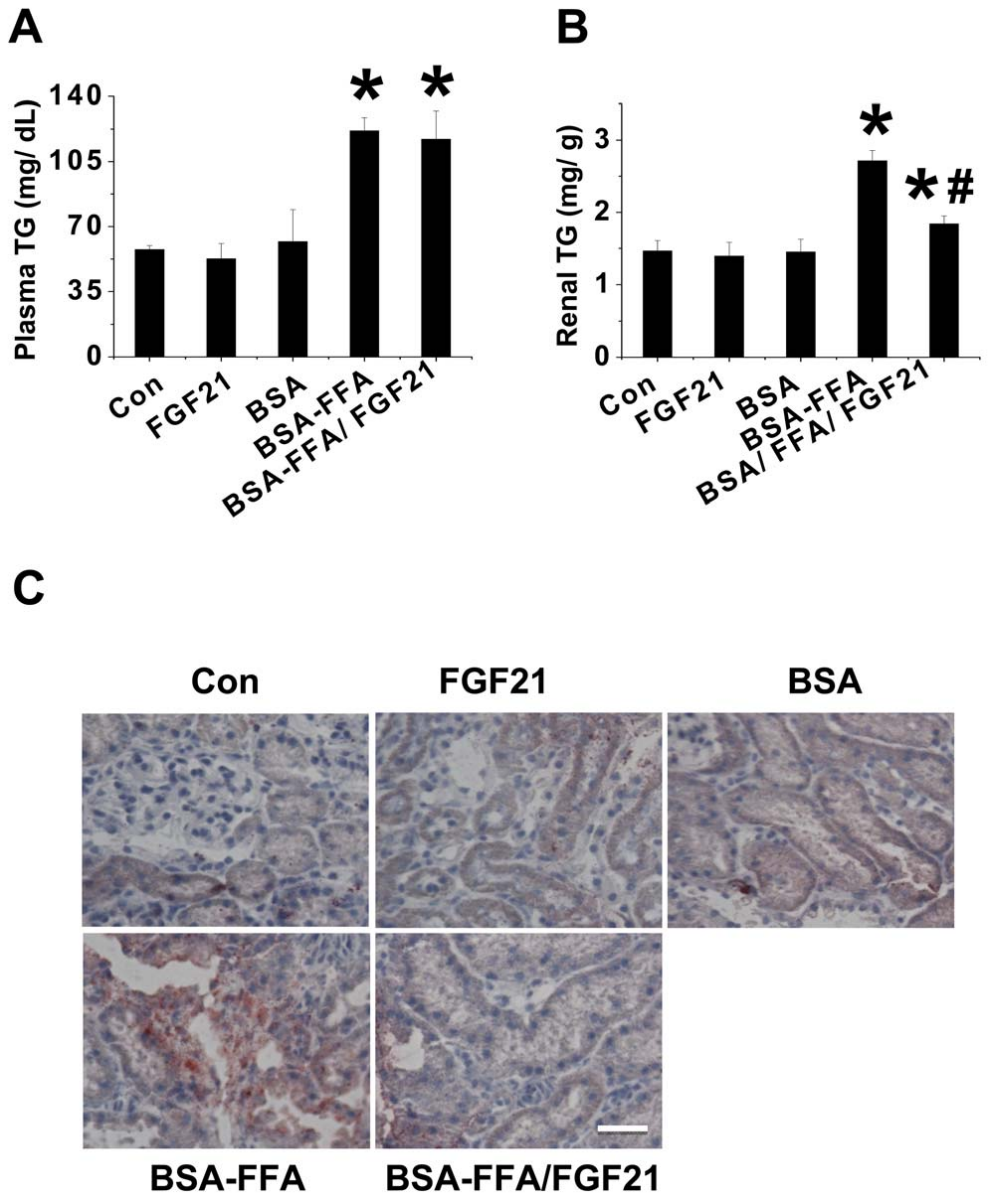
FGF21 prevents FFA-induced renal lipid accumulation. The FFA injection mouse models were prepared as in Fig. 1. The plasma and renal tissue were collected. Plasma TG (A) and renal TG (B) were examined by ELISA kits and TG reagent respectively. Renal lipid accumulation was examined by oil red O staining (C, 40×). Data are presented as mean ± SD (n  =  6 at least in each group). * *P* < 0.05 compared with control. ^#^ *P* < 0.05 compared with BSA-FFA.

**FGF21 prevented FFA induced lipid accumulation in the kidney.** Excessive lipid accumulation is the principle instigator of renal damage caused by lipotoxicity. We found that the administration of FGF21 significantly prevented lipotoxicity-induced renal apoptosis and dysfunction. Therefore, we tried to identify whether the renal protection provided by FGF21 was associated with suppression of renal lipid accumulation. We detected plasma and renal TG levels as well as lipid accumulation in the kidneys of the mice of the treatment groups. The results showed that under normal conditions, neither FGF21 nor BSA had any impact on plasma or renal TG levels, but these were significantly higher after 10 days of BSA-FFA treatments (Fig. 2A, B). FGF21 remarkably lowered FFA-induced renal TG levels but had no impact on plasma TG levels although a decrease tendency was observed (Fig. 2B). In addition, Oil Red O staining also confirmed that FFA significantly increased lipid accumulation in the kidney, which was strongly attenuated by FGF21 treatment (Fig. 2C).

**Figure 3:**
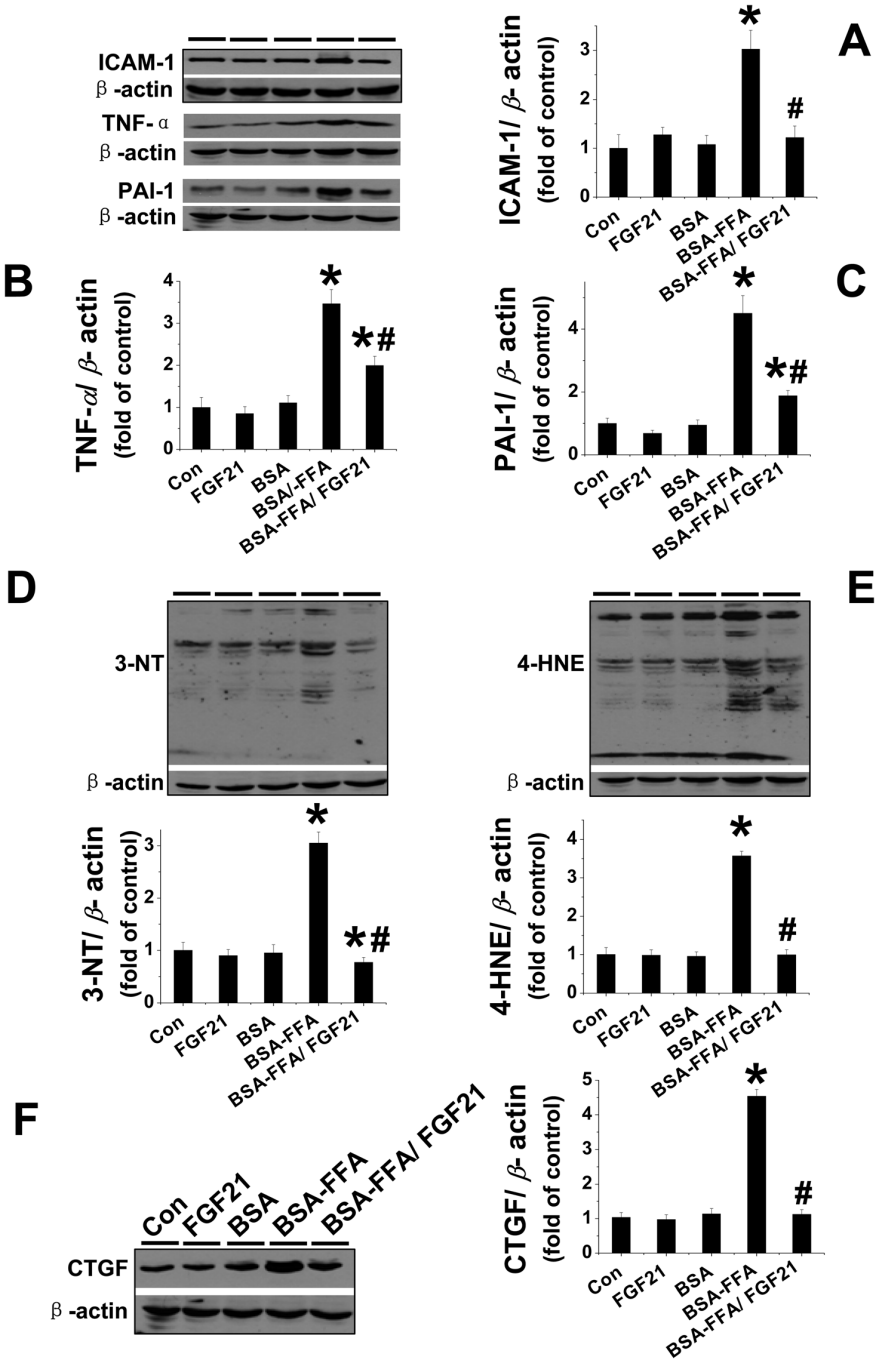
FGF21 prevented FFA-induced inflammation oxidative damage and fibrotic effect. Renal expression of inflammatory factors, including ICAM -1 (A), TNF-α (B), and PAI -1 (C) was examined by Western blotting. Renal oxidative damage was examined by Western blot for the expression of 3-NT as an index of protein nitration (D) and 4-HNE as an index of lipid peroxidation (E). Renal fibrotic effect was measured by Western blot for the expression of CTGF (F). Data are presented as mean ± SD (n  =  6 at least in each group). * *P* < 0.05 compared with control. ^#^ *P* < 0.05 compared with BSA-FFA.

**FGF21 prevented lipotoxicity induced inflammation, oxidative stress and fibrotic effect.** Inflammation is a principle pathological consequence of lipotoxicity, characterized by the release of multiple inflammatory factors. Results of a recent study suggested that FGF21 prevented cardiac hypertrophy via suppression of inflammation [20]. Thus, we next determined whether the renal protective effect associated with FGF21 against lipotoxicity was directly anti-inflammatory. The protein levels of the classic inflammatory factors ICAM-1, TNF-α, and PAI-1 were detected via Western blot assay. The results revealed that FFA strongly upregulated the expressions of ICAM-1, TNF-α, and PAI-1 in the kidney (Fig. 3A-C), but these effects were not observed in either the FGF21 or BSA groups (Fig. 3A-C). Administration of FGF21 almost completely reversed the FFA-induced upregulation of renal ICAM-1 expression from the baseline (Fig. 3A) and significantly attenuated both TNF-α and PAI-1 expression in the kidney (Fig. 3B, C).

**Figure 4:**
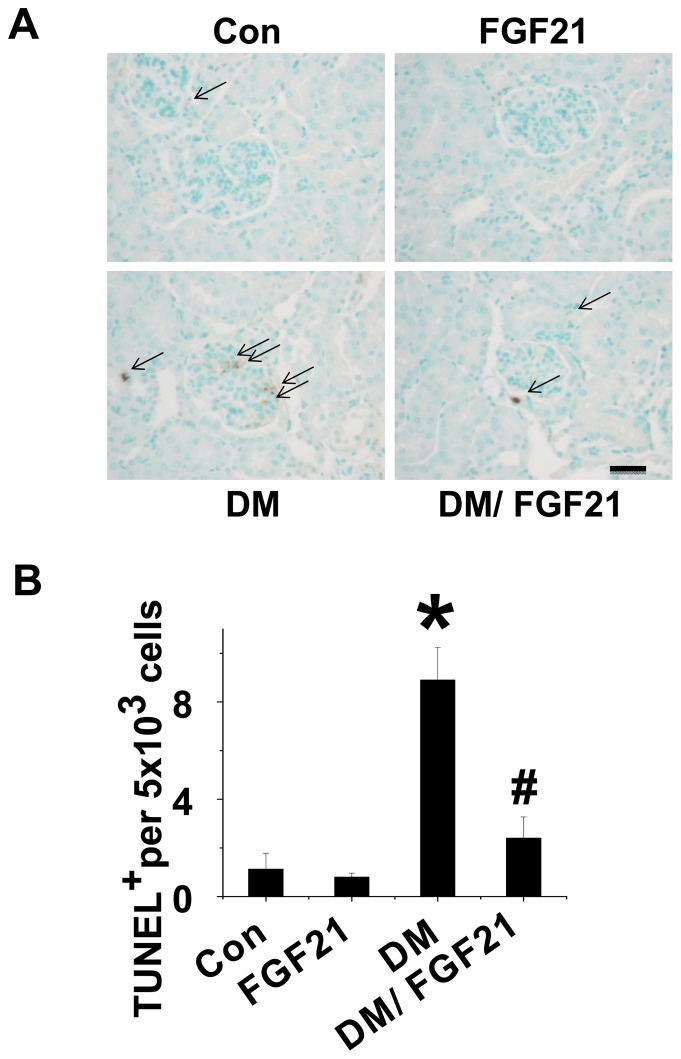
FGF21 prevents diabetes-induced cardiac apoptosis. Type 1 diabetes was induced with STZ (150 mg/kg). Diabetic and age-matched control mice were administered daily intraperitoneal injections of FGF21 (100 µg/kg) or PBS for 10 days. Renal apoptosis was examined with TUNEL staining (A) and semi-quantitative analysis for apoptotic examination was scored (B). Data are presented as mean ± SD (n  =  6 at least in each group). * *P* < 0.05 compared with control. ^#^ *P* < 0.05 compared with diabetes (DM).

Renal inflammatory responses are always associated with oxidative stress and pro-fibrotic effects [34]–[40]. Via Western blot, we determined the effect of FGF21 on lipotoxicity-induced oxidative stress by measuring 3-NT as an index of nitrosative damage (Fig. 3D) and 4-HNE (lipid peroxide) as an index of oxidative damage (Fig. 3E); CTGF, an index of fibrosis, was considered a reflection of the fibrotic effect (Fig. 3F). The results indicated that FFA strongly upregulated 3-NT, 4-HNE, and CTGF expression in the kidney, which were significantly suppressed by FGF21 treatment. However, under normal conditions, neither FGF21 nor BSA had any impact on renal 3-NT, 4-HNE, or CTGF expression (Fig. 3).

### FGF21 prevented diabetes induced kidney disease

**FGF21 prevented renal dysfunction under diabetic conditions.** There is much evidence to support that lipotoxicity is a key component in the pathogenesis of DKD [7]–[9], [41]–[43]. Thus, we next determined whether FGF21 had protective effect against diabetes-induced renal pathological changes, and if so, whether the possible protective mechanism was attributable to an anti-lipotoxic role. FVB mice were intraperitoneally injected with a single high dose of STZ (150 mg/kg) to induce type 1 diabetes. Then diabetic mice and age-matched non-diabetic mice were further stratified into groups with and without treatment of FGF21 (100 µg/kg) daily for 10 days. KW/TL and renal function were determined (Table 2). The results showed that the mouse diabetic model had slightly but not significantly higher KW/TL, PCR, BUN, and ACR compared to the control group. After treatment with FGF21, the tendencies toward higher levels of these indices was no longer present, but were all were at normal levels (Table 2). However, under normal conditions, FGF21 had no impact on the kidney weight or renal function.

**Table 2:** Effect of FGF21 on diabetes-induced renal hypertrophy and dysfunction.

	Control	FGF21	DM	DM/FGF21
KW/TL (mg/mm)	17.63±4.11	17.25±3.07	21.73±4.46	18.98±4.54
PCR (mg/mg)	25.55±4.98	25.65±3.47	27.12±5.21	25.55±4.17
BUN (mg/dl)	36.43±4.23	37.38±5.64	39.49±4.98	37.61±5.15
ACR (µg/mg)	103.36±8.55	107.64±7.17	113.09±10.71	109.12±10.33

Note: DM means diabetes mellitus.

**FGF21 prevented renal apoptosis under diabetic conditions.** Diabetes-induced renal damage usually initiates with tubular cell apoptosis [44]. Thus, we next determined whether FGF21 can protect renal cells from apoptosis induced by diabetes. TUNEL staining was performed in renal tissues of the mice from each group (Fig. 4A, B). Increased positive staining for apoptosis was observed in the kidney of diabetic mice, which was significantly prevented by FGF21 treatment. The combined results of TUNEL-positive cells from 3 independent experiments are quantitatively summarized in Figure 4B, which shows that FGF21 remarkably prevented diabetes-induced renal apoptosis.

**Figure 5:**
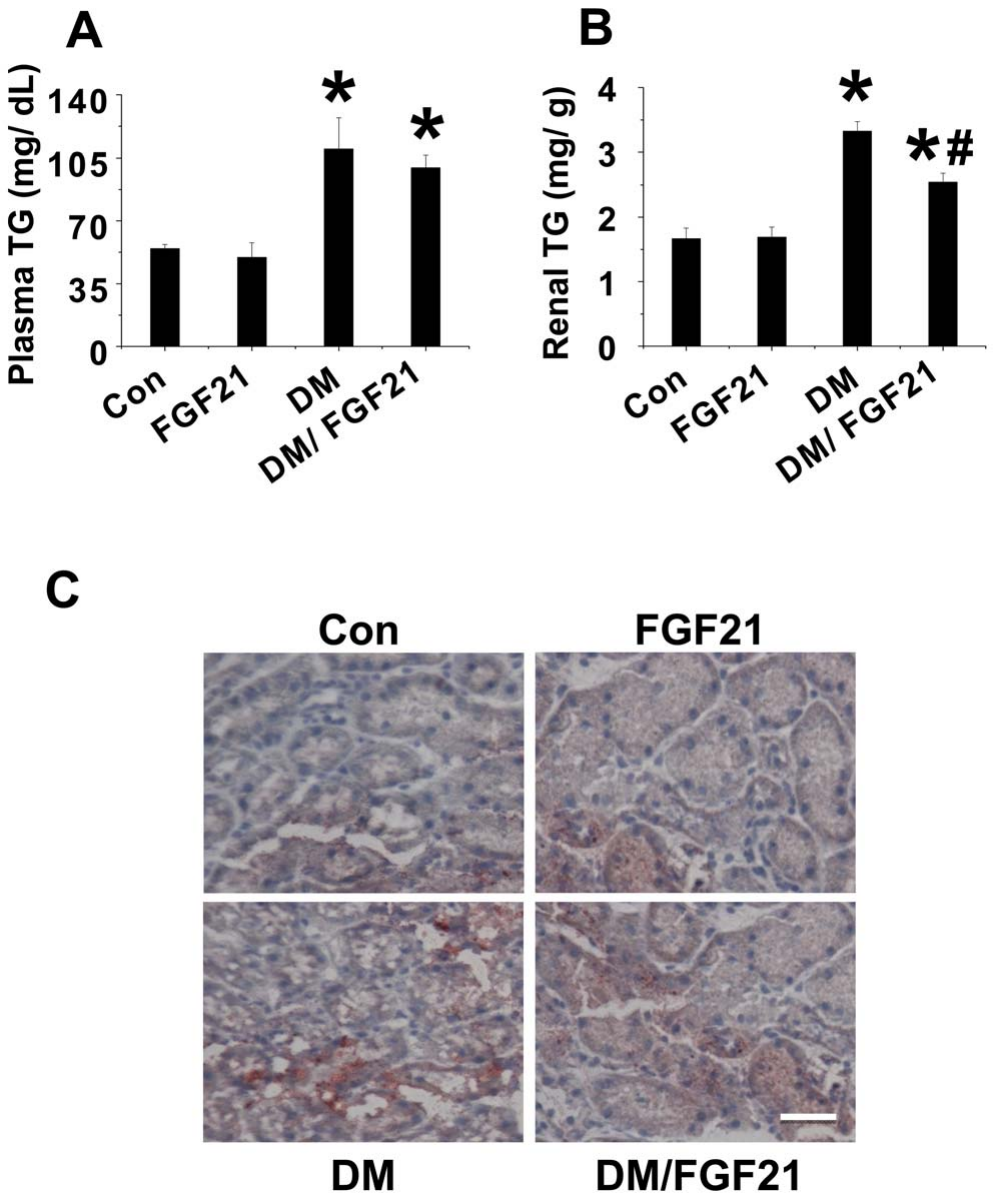
FGF21 prevents diabetes-induced renal lipid accumulation. The diabetic mouse models were prepared as in Fig. 4. The plasma and renal tissue were collected. Plasma TG (A) and renal TG (B) were examined by ELISA kits and TG reagent respectively. Renal lipid accumulation was examined by Oil Red Staining (C, 40 ×). Data are presented as mean ± SD (n  =  6 at least in each group). *P* < 0.05 compared with control. ^#^ *P* < 0.05 compared with diabetes (DM).

**FGF21 prevented diabetes induced lipid accumulation in the kidney.** Compared to the untreated diabetic model, mice treated with FFA is more likely to cause renal lipotoxicity and further amplify the damage signal. Although the renal function of the diabetic mice was only slightly impaired, we still found that FGF21 had a protective effect. Thus whether FGF21 can decrease lipid accumulation in the kidney needed to be determined. We detected the plasma and renal TG levels, and lipid accumulation in the kidneys, of the mice in the different groups. In control group, FGF21 had no impact on either plasma or renal TG levels (Fig. 5A, B), but these were significantly higher in the diabetic mice (Fig. 5A, B). Although FGF21 slightly suppressed diabetes-induced plasma TGs, there was no significant difference between the untreated diabetic model mice and diabetic mice treated with FGF21 (Fig. 5A). In contrast, FGF21 significantly suppressed diabetes-induced elevations in renal TG levels (Fig. 5B). In addition, Oil Red O staining revealed that diabetes strongly increased lipid accumulation in the kidney, which was significantly attenuated by FGF21 treatment (Fig. 4C).

**Figure 6:**
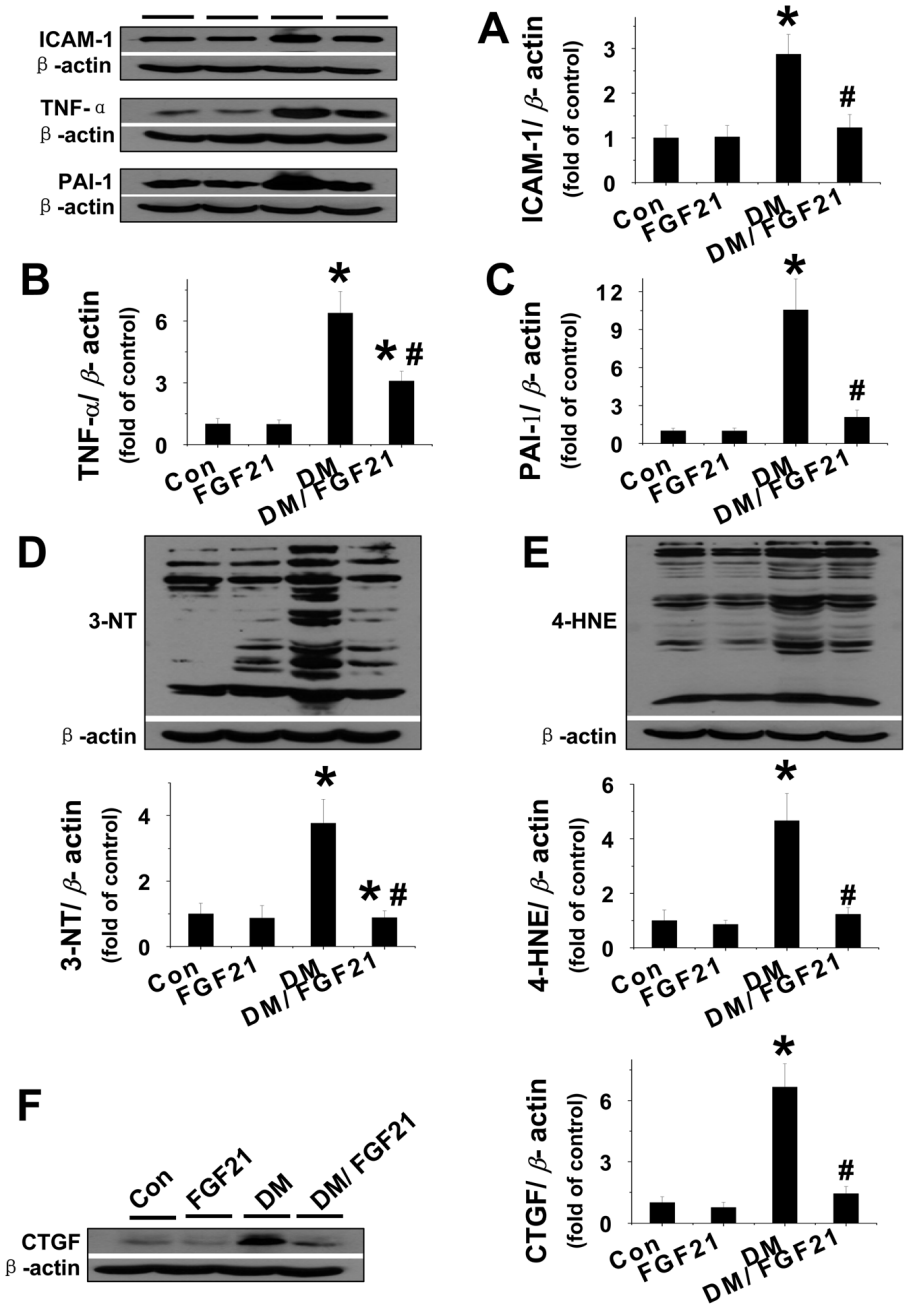
FGF21 prevented diabetes-induced inflammation, oxidative damage and fibrotic effect. Renal expression of inflammatory factors, including ICAM -1 (A), TNF-α(B), and PAI -1 (C) was examined by Western blotting. Renal oxidative damage was examined by Western blotting assay for the expression of 3-NT as an index of protein nitration (D) and 4-HNE as an index of lipid peroxidation (E). Renal fibrotic effect was measured by Western-blotting for the expression of CTGF (F). Data are presented as mean ± SD (n  =  6 at least in each group). * *P*< 0.05 compared with control. *P* < 0.05 compared with control. # *P* < 0.05 compared with diabetes (DM).

**FGF21 prevented diabetes induced inflammation, oxidative stress and fibrotic effect.** Based on our finding that FGF21 prevented lipotoxicity-induced renal inflammation, oxidative stress, and fibrosis we next examined whether FGF21 had a beneficial effect against DKD. The Western blot assay revealed that in normal mice, FGF21 had no influence on the expression of renal ICAM-1, TNF-α, or PAI-1, levels of which were strongly elevated in diabetic mice (Fig. 6A-C). However, administration of FGF21 almost completely suppressed the increased expression of all these inflammatory factors induced by diabetes in the kidney (Fig. 6A-C). Additionally we found that diabetes increased renal 3-NT, 4-HNE, and CTGF expression, which were also significantly suppressed by FGF21 treatment (Fig. 6D-F).

### Deficiency of FGF21 enhanced diabetes induced pathological changes in the kidney

**Deficiency of FGF21 aggravated diabetes-induced chronic renal dysfunction.** In the present study, type 1 diabetes was induced in both C57BL/6J and FGF21-KO mice. Diabetic mice and age-matched non-diabetic mice were divided into groups with and without treatment of FGF21 (100 µg/kg) daily for either 10 days or 3 months. The results showed that diabetes significantly induced kidney weight increase and dysfunction in FGF21-KO mice at the early-stage of diabetes, which was not found in C57BL/6J mice (Table 3). In C57BL/6J mice, a slight increase in KW/TL, PCR, BUN, and ACR became significant at 80 days with diabetes, which was further enhanced in FGF-KO mice (Table 3). However, administration of FGF21 remarkably prevented diabetes-induced renal dysfunction and hypertrophy.

**Table 3:** Renal function under diabetic condition in both C57BL/6J and FGF21-KO mice.

		KW/TL (mg/mm)		PCR (mg/mg)		BUN (mg/dl)		ACR (µg/mg)	
		10 d	80 d	10 d	80 d	10 d	80 d	10 d	80 d
Control	C57BL/6J	18.06±3.91	18.55±4.14	25.65±4.32	24.32±4.28	37.83±3.58	31.69 ±4.39	106.96±5.15	112.97±10.49
	FGF21-KO	18.44±3.79	19.86±4.13	24.14±3.98	24.73±4.45	36.99±4.87	31.64±3.36	107.12±5.64	110.92±9.47
DM	C57BL/6J	21.66±3.21	27.15±2.92	28.46±3.23	201.44±14.32	39.14±5.15	49.93±5.75	114.77±7.64	21324.32±121.68
	FGF21-KO	26.13±3.59	35.65±1.35 a,b	103.44±9.85	253.43±15.38 a,b	47.92±4.61	61.11±9.58 a,b	10986.36±98.91	28974.13±115.67 a,b
STZ	C57BL/6J	18.54±4.14	18.64±1.52	24.75±4.87	24.84±3.19	37.11±5.19	31.43±3.54	104.63±7.72	114.45±9.64
	FGF21-KO	18.87±3.19	19.51±4.6	25.12±4.33	23.39±4.69	36.23±3.97	31.65±4.38	106.05±5.07	113.44±12.79
DM/FGF21	FGF21-KO	19.64±4.01	21.89±2.79 ^c^	25.35±3.71	101.32±10.79 a,c	37.31±5.41	41.16±4.29 a,c	108.15±9.04	18008.33±126.47 a,c

a *P* < 0.05 compared with corresponding control; ^b^ *P* < 0.05 compared with DM in C57BL/6J mice; ^c^ *P* < 0.05 compared with corresponding DM.

**Figure 7:**
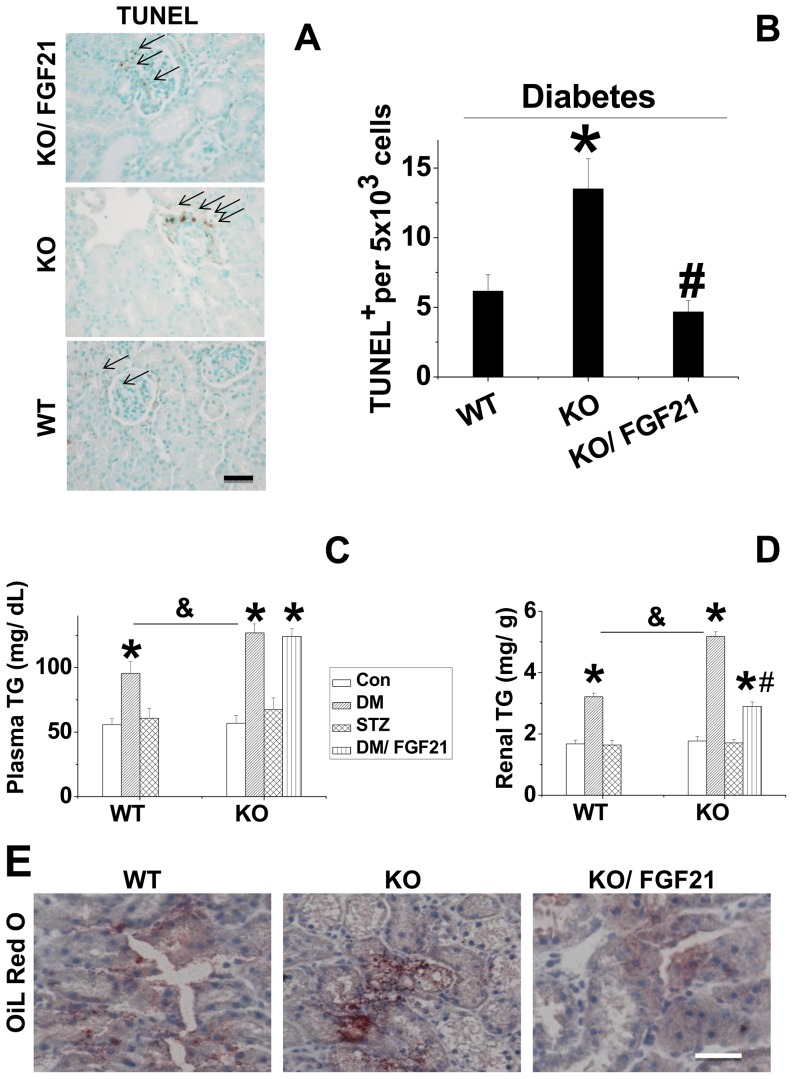
FGF21-KO mice were more sensitive to diabetes-induced cardiac apoptosis and lipid accumulation. FGF21-KO and C57BL/6J (C57BL/6J) mice were induced as diabetic with STZ (200 mg/kg) and treated with FGF21 (100 µg/kg) for 10 days. Renal apoptosis was detected with TUNEL staining (A). Quantitative data presented as B. The plasma and renal tissue were collected. Plasma TG (C) and renal TG (D) were examined by ELISA kits and TG reagent respectively. Renal lipid accumulation was examined by Oil Red Staining (E, 40 ×). Data are presented as mean ± SD (n  =  6 at least in each group). *P*< 0.05 compared with corresponding control. # *P* < 0.05 compared with corresponding diabetes (DM). & *P* < 0.05 compared with DM in wild type group.

**Deficiency of FGF21 aggravated diabetes induced renal lipid accumulation and apoptosis.** Renal apoptosis and lipid accumulation were examined and compared between C57BL/6J and FGF21-KO mice under either the normal or diabetic condition. We found in the C57BL/6J mice that diabetes, but not STZ, strongly induced renal apoptosis, which was further enhanced in FGF21-KO mice. Administration of FGF21 significantly protected renal cells from apoptosis induced by diabetes (Fig. 7A, B). Furthermore, we found that the diabetes, rather than STZ, significantly increased both plasma and renal TG levels in C57BL/6J mice, which was further aggravated in FGF21-KO mice (Fig. 7C, D). Administration of FGF21 had no influence on plasma TG levels (Fig. 7C), but showed great lowering effect on renal TG levels (Fig. 7D). Oil Red O staining confirmed that deficiency of FGF21 further enhanced diabetes-induced lipid accumulation in the kidney, which was significantly attenuated by administration of FGF21 (Fig. 7E).

**Figure 8:**
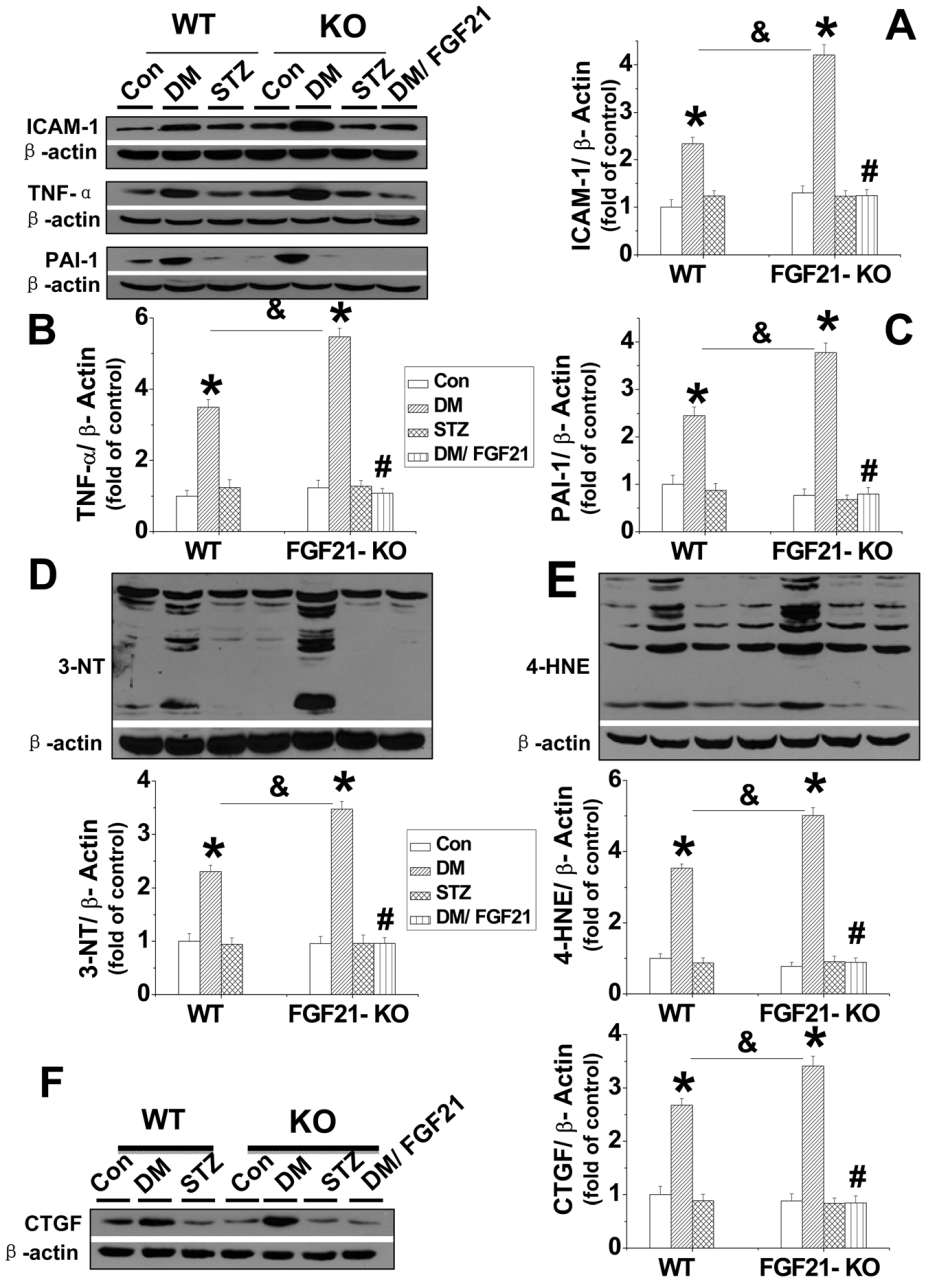
FGF-21 KO further enhances diabetes induced renal inflammation, oxidative damge and fibrotic effect. Renal expression of inflammatory factors, including ICAM -1 (A), TNF (B), and PAI -1 (C) was examined by Western blotting. Renal oxidative damage was examined by Western blotting assay for the expression of 3-NT as an index of protein nitration (D) and 4-HNE as an index of lipid peroxidation (E). Renal fibrotic effect was measured by Western-blotting for the expression of CTGF (F). Data are presented as mean ± SD (n  =  6 at least in each group). *P*< 0.05 compared with corresponding control. # *P* < 0.05 compared with corresponding diabetes (DM). & *P* < 0.05 compared with DM in wild type group.

**Deficiency of FGF21 aggravated diabetes-induced inflammation, oxidative stress, and fibrotic effect.** Western-blot assay revealed that diabetes, but not STZ significantly upregulated ICAM-1, TNF-α, and PAI-1 expression in the kidneys of C57BL/6J mice, which were further increased in FGF21-KO mice (Fig. 8A-C). Deficiency of FGF21 also further enhanced diabetes-induced oxidative stress and fibrotic effect by upregulation of the expression of renal 3-NT/4HNE and CTGF (Fig. 8 D-F). Administration of FGF21 greatly attenuated diabetes-induced renal inflammation, oxidative stress, and fibrotic effect in FGF21-KO mice (Fig. 8A-F).

## Discussion

In the present study, the lipotoxic mouse model was induced by intraperitoneal injection of FFA bound to BSA (10 mg/g) as described previously [27], [28]. In this model, FFA bound to albumin had a role in the generation of tubulointerstitial disease associated with the release of inflammatory factors, which in turn enhanced tubulointerstitial damage. In the present study, acute injection of FFA induced renal dysfunction, characterized by an increase in PCR and ACR. Furthermore, the kidney weight increased in FFA-treated mice, which was a sign of renal hypertrophy. These renal pathological changes indicated that the lipotoxic mouse model was successfully established (Table 1). We also found that administration of FGF21 significantly prevented lipotoxicity induced renal hypertrophy and dysfunction. It is generally accepted that renal dysfunction always initiates with apoptosis [44]. Thus, in the present study we determined whether the renal beneficial effect induced by FGF21 against lipotoxicity was attributed to anti-apoptosis. TUNEL staining showed that FFA injection significantly induced apoptosis in the kidney, but a similar phenomenon was not found in BSA-treated mice, and this apoptosis was remarkably attenuated by administration of FGF21. This result confirmed that FGF21-induced renal protection against lipotoxicity was due to an anti-apoptotic effect.

In the present study, we found for the first time that FGF21 has a crucial role in renal protection against lipotoxicity by preventing renal apoptosis and dysfunction. Subsequent to this finding, we attempted to determine the underlying mechanisms of this protective effect. Previously we found that FGF21 lowered cellular lipid accumulation through enhancement of fatty acid oxidation and lipolysis. Therefore we had to identify whether the lipid-lowering effect attributed to FGF21 induced renal protection. We determined the renal TG levels and lipid accumulation among the treatment groups, and found FFA significantly increased lipid accumulation and TG levels in kidney tissues, which was remarkably suppressed by administration of FGF21. However, no similar phenomenon was found in plasma TG levels. This implies that FGF21-induced renal protection was specifically due to a lipid-lowering effect in the kidney rather than through systematic lipid metabolism. Simultaneously we found that FGF21 significantly, but not completely, lowered renal lipid accumulation, suggesting that FGF21 induced renal protection only partially by lessening renal lipid accumulation. Other protective mechanisms are probable.

The inflammatory response is considered one of the major mechanisms by which lipotoxicity causes renal oxidative injury, fibrosis, and dysfunction [45]-[48]. This process includes diverse inflammatory molecules, for example, increases in ICAM-1 lead to inflammatory cell migration in renal inflammatory pathogenesis [49]. TNF-α contributes significantly to sodium retention and renal hypertrophy and alters the barrier function of the glomerular capillary wall, resulting in an enhanced albumin permeability [34], [35], [49].

In our study, we found significant increases in the renal expression of the proinflammatory cytokines ICAM-1, TNF-α, and PAI-1 induced by FFA injection. A consequence of the inflammatory response is the overgeneration of reactive oxygen species that induces oxidative and nitrosative damage characterized by increased renal accumulation of 3-NT and 4-HNE [50], [51]. Our study also confirmed that FFA strongly upregulated the expressions of renal 3-NT and 4-HNE. Moreover, since PAI-1 also promotes collagen deposition by stimulating migration of leukocytes and collagen-producing cells into damaged tissues finally leading to fibrosis [38], [52], it is also considered a fibrotic factor. Analysis of another fibrotic marker, CTGF, in the kidney also proved that lipotoxicity strongly induced fibrosis as well as elevating inflammation and oxidative stress. However, all the above pathological changes induced by lipotoxicity were significantly prevented by FGF21 treatment. This suggests that FGF21-induced renal protection against acute lipotoxicity was not only due to lowering lipid accumulation but also to the inhibition of subsequent inflammation, oxidative stress, and fibrotic effect in the kidney.

The kidney is one of the main organs damaged by diabetes, leading to DKD and subsequent diabetic nephropathy, and accompanied by an increased risk of cardiovascular disease [53], decreased quality of life, increases in financial costs to the patient and society, and shortened life span [53]. Since lipotoxicity is a key pathogenic cause of DKD [7]-[9], [41], we investigated whether FGF21 can induce similar renal protection in a diabetic model as in the FFA injection model, and if so, whether this protection was associated with attenuation of diabetic lipotoxicity and subsequent inflammation, oxidative stress, and fibrotic effect. To answer these questions, type 1 diabetic mice induced by STZ and age-matched non-diabetic mice were treated with or without FGF21 for either 10 days or 80 days. We found at the early stage (10 days) that, although renal hypertrophy and dysfunction was not observed, diabetes significantly induced renal apoptosis and an increase of lipid accumulation and subsequent inflammation, oxidative stress, and fibrotic effect, which were remarkably prevented by FGF21 treatment. In contrast, at 80 days renal hypertrophy and dysfunction were observed in the diabetic mice, evidenced by increases in BUN, PCR and ACR. Administration of FGF21 significantly prevented renal damage-induced diabetes. This indicates that FGF21 induced renal protection against early-stage renal apoptosis and later-stage renal dysfunction due to diabetes, through prevention of lipid accumulation and subsequent inflammation, oxidative stress, and fibrotic effect.

We also found that diabetes induced renal lipotoxicity, and the subsequent pathological changes were further enhanced in FGF21-KO mice. Similar phenomena were not found in STZ-treated mice which unsuccessfully developed hyperglycemia. Administration of FGF21 significantly prevented renal damage induced by diabetes. This suggests that all the pathological changes were induced by diabetes, and not by STZ per se. In addition, FGF21-KO mice were more sensitive to diabetes-induced renal injury, which was remarkably prevented by FGF21 treatment through the mechanism of lowering lipid accumulation, and by anti-inflammation, anti-oxidation, and anti-fibrosis effects.

Mechanistically the beneficial effect of FGF21 against oxidative stress, inflammation and fibrosis may related to the activation of adenosine 5‘-monophosphate -activated protein kinase (AMPK) induced signaling pathway [54]. AMPK is a crucial kinase in eukaryotes, which induced multiple bio-functions by activation of the downstream Sirtuin (SirT)1- peroxisome proliferator activated receptor co-activator (PGC) 1α signaling pathway [7], [54]. Increasing evidence showed that activation of AMPK-SirT1-PGC1α pathway prevented inflammation and oxidative stress through inhibiting NF-κB function and promoting fatty-acid β-oxidation as well as antioxidant expressions [55]–[60]. Moreover, it is reported that AMPK also participated in anti-fibrosis by inhibiting TGF-β1 to ameliorate renal fibrosis and structure alterations [61]. Reportedly AMPK signaling pathway can be activated by FGF21 through up-regulating the expression of AMPK activator liver kinase B1 (LKB1) [62], which implied that AMPK may be the mediator for FGF21-induced anti-oxidative stress, anti-inflammation, and anti-fibrosis in the kidney under diabetic conditions.

In summary, we report for the first time that administration of FGF21 can significantly prevent lipotoxicity- and diabetes-induced early-stage renal apoptosis, hypertrophy, and dysfunction, and significant prevented renal lipid accumulation and subsequent inflammation, oxidative damage and fibrotic effect. Deficiency of FGF21 (in FGF21-KO mice) enhanced lipotoxicity and diabetes-induced renal damages, which were significantly prevented by intraperitoneal injection of FGF21. Therefore, our study suggests that FGF21 is a potential candidate for therapeutic application against DKD.
